# Taking the inner route: spatial and demographic factors affecting vulnerability to COVID-19 among 604 cities from inner São Paulo State, Brazil

**DOI:** 10.1017/S095026882000134X

**Published:** 2020-06-19

**Authors:** C. M. C. B. Fortaleza, R. B. Guimarães, G. B. de Almeida, M. Pronunciate, C. P. Ferreira

**Affiliations:** 1 Department of Infectious Diseases, Botucatu School of Medicine, São Paulo State University (UNESP), City of Botucatu, São Paulo State, Brazil; 2 Department of Geography, Faculty of Science and Technology, São Paulo State University (UNESP), City of Presidente Prudente, São Paulo State, Brazil; 3 Department of Biostatistics, Botucatu Institute of Biosciences, São Paulo State University (UNESP), City of Botucatu, São Paulo State, Brazil

**Keywords:** COVID-19, epidemiology, ecologic study, virus infection

## Abstract

Even though the impact of COVID-19 in metropolitan areas has been extensively studied, the geographic spread to smaller cities is also of great concern. We conducted an ecological study aimed at identifying predictors of early introduction, incidence rates of COVID-19 and mortality (up to 8 May 2020) among 604 municipalities in inner São Paulo State, Brazil. Socio-demographic indexes, road distance to the state capital and a classification of regional relevance were included in predictive models for time to COVID-19 introduction (Cox regression), incidence and mortality rates (zero-inflated binomial negative regression). In multivariable analyses, greater demographic density and higher classification of regional relevance were associated with both early introduction and increased rates of COVID-19 incidence and mortality. Other predictive factors varied, but distance from the State Capital (São Paulo City) was negatively associated with time-to-introduction and with incidence rates of COVID-19. Our results reinforce the hypothesis of two patterns of geographical spread of SARS-Cov-2 infection: one that is spatial (from the metropolitan area into the inner state) and another which is hierarchical (from urban centres of regional relevance to smaller and less connected municipalities). Those findings may apply to other settings, especially in developing and highly heterogeneous countries, and point to a potential benefit from strengthening non-pharmaceutical control strategies in areas of greater risk.

## Introduction

The impact of COVID-19 in metropolitan areas and highly populated cities has been addressed by both surveillance data and mathematical models. Based on suboptimal evidence, public health officials have recommended non-pharmaceutical strategies, such as social distancing [1–3]. The evidence for COVID-19 control measures in smaller cities is even scarcer [4]. This is a major challenge for countries such as Brazil, which are both huge and heterogeneous in socio-economic indexes, demography and access to health care [5].

São Paulo is the most populous and richest State in Brazil, with a population of 44 million inhabitants. Half of those people live in São Paulo Metropolitan area, a cluster of cities with high level of conurbation and demographic density. The impact of COVID-19 on that area was predicted by mathematical models [6], which lead to the implementation of a social distancing strategy for the whole state since 23 March 2020.

Preliminary studies demonstrated that this strategy lowered SARS-Cov-2 transmission and reproductive number of COVID-19 in the metropolitan area [7]. However, data on population mobility, identified by spatial monitoring of mobile phones (available at São Paulo State Government site, https://www.saopaulo.sp.gov.br/coronavirus/isolamento/) have detected lower adherence to social distancing in inner municipalities of the State. Also, the same system has demonstrated a trend towards neglecting governmental recommendations all over the state.

Many countries are now facing the exhaustion of lockdown measures and planning the return of social and economic activities [8, 9]. In a state that is more populous than several countries, any strategy for loosening social distancing measures must be carefully guided by epidemiological data and a rigorous assessment of regional risk. Therefore, we conducted a study aimed at identifying factors that affect vulnerability to COVID-19 among 604 municipalities in São Paulo State that are located outside the capital metropolitan area metropolitan area. We also aimed at providing a methodological approach that may be useful for other countries.

## Methods

### Study setting, design and exclusion criteria

São Paulo State has 645 municipalities and 46 million inhabitants. Our study included 604 municipalities from inner São Paulo State, Brazil, with aggregate population of ~24 million inhabitants. As of 8 May 2020, 247 (40.9%) municipalities did not report any case, and 357 (59.1%) of those municipalities reported 7181 confirmed cases and 488 deaths due to COVID-19. Since we were interested in studying the spread of COVID-19 in the inner State, we excluded from our analysis the state capital and cities located in its metropolitan area.

### Data collection and analysis

**Fig. 1.**
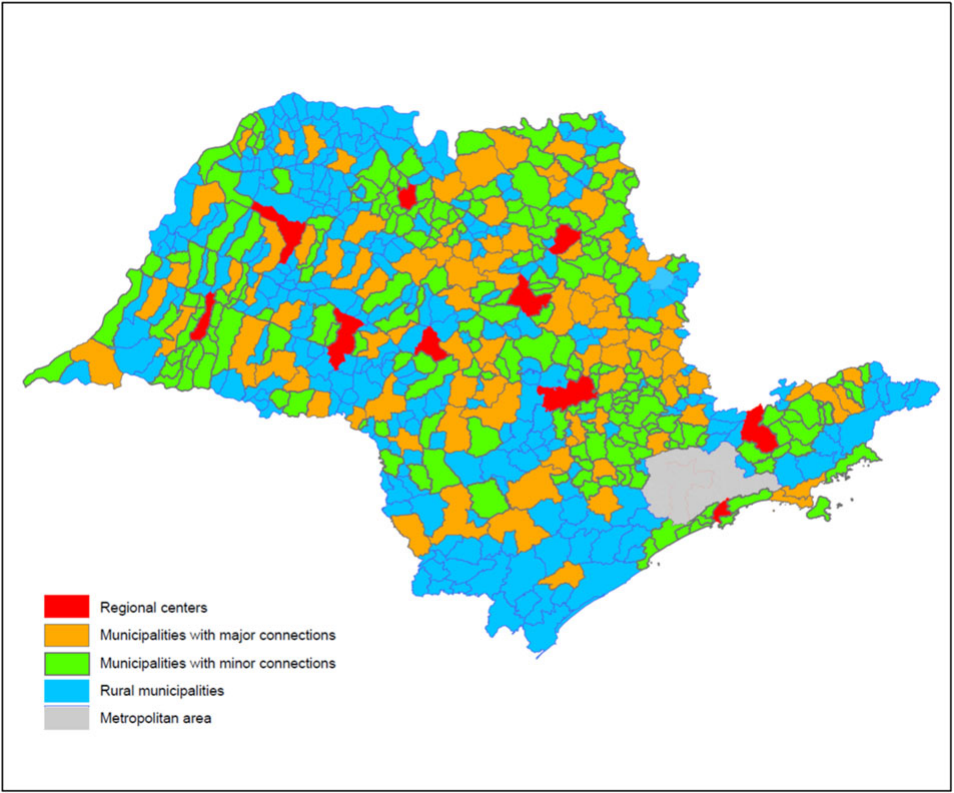
Map of São Paulo State, Brazil, highlighting different classifications of municipalities, according to their regional influence and connectiveness. The São Paulo City metropolitan area (grey area) was excluded from our analysis.

Data on notifications of confirmed COVID-19 cases and deaths were obtained from the Centre for Epidemiological Surveillance from São Paulo, State Health Department (CVE, www.cve.saude.sp.gov.br). Socio-demographic data for each municipality were obtained from the São Paulo State Foundation for Data Analysis (SEADE, https://www.seade.gov.br/). These data included demographic density, percentage of persons living in urban area, human development index (HDI) and Gini index for inequality of income [10]. Based on our previous analysis [11], we identified 13 municipalities which are centres of greater regional influence. The remaining municipalities were classified according to criteria from the Brazilian Institute for Geography and Statistics (2017) [12]: (a) urban municipalities under major influence from regional centres; (b) urban municipalities under minor influence from regional centres; (c) rural municipalities (Fig. 1). In all models, the 13 regional centres were used as reference category. Finally, we identified the road distance from each municipality to the State capital, São Paulo City (http://www.cidadespaulistas.com.br/prt/cnt/distancias.html).

### Statistical analysis

A descriptive analysis of data was performed to identify differences for major categories of municipalities. In the following step, we used multivariable Cox regression models to analyse time from the first report of COVID-19 in São Paulo State up to the first occurrence of autochthonous case in each municipality. We performed both univariate and multivariable analysis (including simultaneously all predictors cited above). Similarly, we also performed univariate and multivariable models of zero-inflated binomial negative regression, with rates of laboratory-confirmed cases and mortality due to COVID-19 as outcomes. The same variables were assessed as risk factors. All analyses were performed using STATA 14 (Statacorp, College Station, TX, USA) or SPSS22 (IBM, Armonk, NY, USA).

## Results

**Fig. 2.**
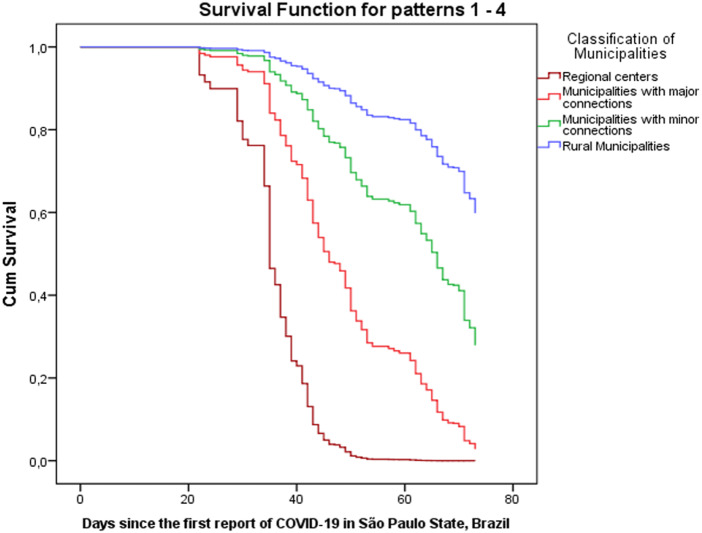
Cox regression graphics for time until introduction of COVID-19 in municipalities from inner São Paulo State, Brazil (based on surveillance data up to 8 May 2020).

Early occurrence and both higher incidence and mortality rates were reported in municipalities classified as major regional centres (Table 1). In univariate Cox regression, several variables were positively associated with early introduction of COVID-19: higher classification of influence/connectiveness, demographic density, proportion of persons living in urban area, HDI and the Gini index for inequalities in income. In an opposite way, distance from the capital had a protective effect (i.e. was negatively associated with the outcome). In multivariable models, influence/connectiveness, demographic density and HDI were predictors of early outcome, while distance from the capital was once again negatively associated (Table 2 and Fig. 2).

**Table 1.** Characteristics of municipalities from inner São Paulo State, Brazil

Types of municipalities	Number	Total population	Days until 1st case	COVID-19 incidence^a^	COVID-19 mortality^a^
Regional centres	13	5 953 766	22	42.5 (32.4–49.5)	2.1 (1.4–3.0)
Municipalities under major influence	87	6 993 951	31	12.4 (6.8–28.1)	0.3 (0.0–2.6)
Municipalities under minor influence	194	8 346 815	34	10.6 (0.0–23.9)	0.0 (0.0–1.4)
Rural municipalities	308	2 797 404	55	10.66 (0.0–16.45)	0.0 (0.0–0.0)^b^

a Per 100 000 inhabitants, as of 8 May 2020.

b Though 75th percentiles were zero, upper values for COVID incidence and mortality in rural municipalities were 134.3 and 14.3 per 100 000 inhabitants, respectively. Those were obvious outliers.

**Table 2.** Multivariable Cox regression results for likelihood of inner São Paulo State municipalities presenting at least one confirmed case of COVID-19 as of 8 May 2020

	Univariate analysis		Multivariable analysis	
Predictors	HR (95% CI)	*P*	HR (95% CI)	*P*
Classification of municipalities				
Regional centres	1.00 (reference)	–	1.00 (reference)	–
Municipalities with major connections	0.24 (0.13–0.44)	<0.001	0.32 (0.17–0.59)	<0.001
Municipalities with minor connections	0.12 (0.07–0.22)	<0.001	0.16 (0.09–0.30)	<0.001
Rural municipalities	0.09 (0.05–0.16)	<0.001	0.15 (0.08–0.28)	<0.001
Demographic density (for 100/km^2^ increase)	1.09 (1.07–1.11)	<0.001	1.08 (1.05–1.10)	<0.001
Proportion of persons living in urban areas	1.02 (1.01–1.03)	<0.001	1.01 (0.99–1.02)	0.11
HDI (for 10% increase)	2.10 (1.70–2.44)	<0.001	1.42 (1.16–1.73)	0.01
Gini index for inequalities in income (for 10% increase)	1.22 (1.12–1.41)	0.001	2.20 (0.42–11.34)	0.35
Distance from the State Capital (for 100 km increase)	0.91 (0.86–0.96)	<0.001	0.93 (0.88–0.98)	0.007

HR, hazard ratio; CI, confidence interval.

*Note*: Urbanisation rate is the measure of proportion of inhabitants living in urban area.

In the models of zero-inflated negative binomial regression (both univariate and multivariable), there was a positive association of COVID-19 incidence with higher influence/connectiveness degree, demographic density, HDI and Gini index, and a negative association with distance from the State Capital. The proportion of persons living in urban areas was positively associated with that outcome in univariable, but lost statistical significance after adjusting for confounders (Table 3).

**Table 3.** Zero-inflated negative binomial regression results for rates of confirmed COVID-19 cases in inner São Paulo State municipalities, as of 8 May 2020

	Univariate analysis		Multivariable analysis	
Predictors	HR (95% CI)	*P*	HR (95% CI)	*P*
Classification of municipalities				
Regional centres	1.00 (reference)	–	1.00 (reference)	–
Municipalities with major connections	0.39 (0.21–0.74)	0.004	0.29 (0.13–0.65)	0.003
Municipalities with minor connections	0.33 (0.18–0.61)	<0.001	0.18 (0.09–0.40)	<0.001
Rural municipalities	0.32 (0.17–0.59)	<0.001	0.18 (0.08–0.39)	<0.001
Demographic density (for 100/km^2^ increase)	1.07 (1.04–1.11)	<0.001	1.04 (1.01–1.09)	0.01
Proportion of persons living in urban areas	1.01 (1.01–1.02)	0.005	1.01 (0.99–1.02)	0.30
HDI (for 10% increase)	1.63 (1.32–2.10)	<0.001	0.54 (0.43–0.66)	0.001
Gini index for inequalities in income (for 10% increase)	1.52 (1.32–1.71)	<0.001	1.33 (1.15–1.53)	0.02
Distance from the State Capital (for 100 km increase)	0.85 (0.81–0.90)	<0.001	0.89 (0.84–0.95)	0.001

IRR, incidence rate ratio; CI, confidence interval.

*Note*: Urbanisation rate is the measure of proportion of inhabitants living in urban area.

Univariate results for COVID-19 mortality were similar to those found for incidence. However, in the multivariable analysis, only the higher degrees of influence/connectiveness, demographic density and HDI were significantly and positively associated with increased mortality (Table 4).

**Table 4.** Zero-inflated negative binomial regression results for COVID-19 mortality in inner São Paulo State municipalities, as of 18 April 2020

	Univariate analysis		Multivariable analysis	
Predictors	HR (95% CI)	*P*	HR (95% CI)	*P*
Classification of municipalities				
Regional centres	1.00 (reference)	–	1.00 (reference)	–
Municipalities with major connections	0.53 (0.30–0.94)	0.03	0.53 (0.29–0.99)	0.047
Municipalities with minor connections	0.51 (0.29–0.88)	0.02	0.31 (0.17–0.55)	<0.001
Rural municipalities	0.29 (0.16–0.56)	<0.001	0.16 (0.08–0.32)	<0.001
Demographic density (for 100/km^2^ increase)	1.06 (1.03–1.09)	<0.001	1.07 (1.03–1.11)	<0.001
Proportion of persons living in urban areas	1.03 (1.01–1.05)	<0.002	1.00 (0.97–1.02)	0.95
HDI (for 10% increase)	1.71 (1.20–2.11)	0.002	0.60 (0.46–0.73)	<0.001
Gini index for inequalities in income (for 10% increase)	1.62 (1.33–1.91)	<0.001	1.42 (0.90–1.83)	0.06
Distance from the State Capital (for 100 km increase)	0.88 (0.81–0.96)	<0.001	0.90 (0.90–1.10)	0.97

IRR, incidence rate ratio; CI, confidence interval.

*Note*: Urbanisation rate is the measure of proportion of inhabitants living in urban area.

## Discussion

The COVID-19 pandemic has imposed to epidemiologists the rapid performance of complex analyses, aimed at directing public health policies. In developing countries, such as Brazil, the challenges range from access to healthcare to the devastating effects of economic recession [13–15]. To date, more than half COVID-19 cases in São Paulo State occurred in the capital (São Paulo City) metropolitan area. This has created a false sensation of safety in the inner cities. Local authorities (e.g. city mayors) are questioning social distancing recommendations. Our study was conducted aiming to influence São Paulo Health Department (and ultimately, the State Governor's) policies. Alongside with previous analyses [11], we attempted to identify the routes of spread and the vulnerability of municipalities to COVID-19.

One must notice that inner São Paulo State is heterogeneous, comprising cities that range from 1000 to 1.2 million inhabitants. Our findings highlight the importance of regionally relevant urban centres, some of which are located far from the capital (Fig. 1). Interestingly, both the classification of regional relevance and the demographic density were independently associated with early introduction and higher incidence rates.

It is worth noting that, besides regional relevance and other indexes of urbanisation, proximity to the state capital (i.e. the State epicentre of COVID-19) was also independently associated with early and greater impact of the pandemics. Therefore, we detected two patterns of epidemic spread into the inner state. In one pattern, the disease spreads by contiguity into areas neighbouring the capital and its metropolitan area. In the other, it disseminates rapidly to great cities located in central and western areas of the state, from which it spreads into smaller municipalities. The greater the connectiveness of those municipalities with their regional centres, the greater their vulnerability to COVID-19. This explains the apparent paradox of some high-risk cities being located far from the state capital, and vice versa. On the other hand, lower mortality in cities with higher HDI may reflect difficulties of access to hospitals and emergency centres in poorer municipalities in inner São Paulo State.

Our study may have inaccuracies inherent to the analysis of partial data in an ongoing pandemic, including underreporting and delays in information systems. Some variables presented collinearity (e.g. demographic density and proportion of persons living in urban areas), which were adjusted in the multivariable models. Despite its limitations, the ecological design provides the appropriate results to guide public health decisions [16]. If we are to enter a ‘post-lockdown’ period, it must be planned considering the routes of COVID-19 spread into inner areas of the countries. Late introduction and lower (up-to-date) incidence may be erroneously interpreted as absence of risks. In our perspective, the findings of this study should be interpreted in the opposite way, i.e. suggesting that control measures should be strengthened in urban areas of great social and economical influence, and secondarily in municipalities with major connections with those cities [11]. By identifying target areas for interrupting transmission, countries and states can make fine adjustments in their way out of lockdown or other social distancing strategies.

## Data Availability

The authors state that the database used in the analyses can be available as a supplementary file to the paper, or provided to interested researchers upon reasonable request.
